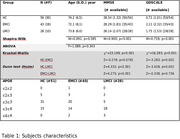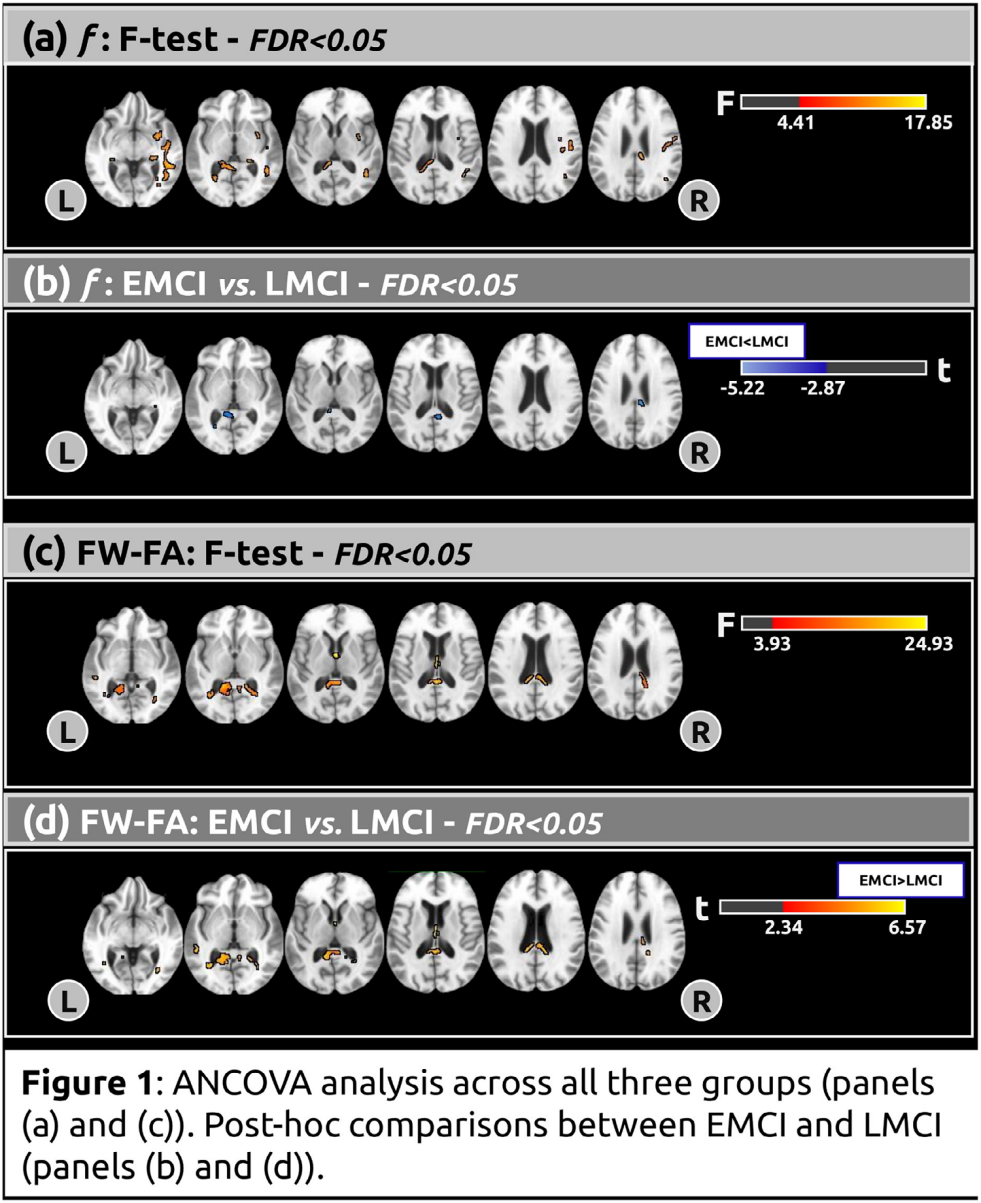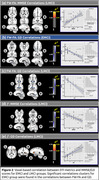# Distinguishing Early Mild Cognitive Impairment from Late Mild Cognitive Impairment through Free‐Water Diffusion Tensor Imaging: A Comparative Analysis

**DOI:** 10.1002/alz.084580

**Published:** 2025-01-09

**Authors:** Maurizio Bergamino, Molly M. McElvogue, Ashley M. Stokes

**Affiliations:** ^1^ Barrow Neurological Institute, Phoenix, AZ USA

## Abstract

**Background:**

Mild Cognitive Impairment (MCI) represents an intermediate stage between normal age‐related cognitive decline and more severe degenerative conditions such as Alzheimer's disease. Understanding the differences between Early‐MCI (EMCI) and Late‐MCI (LMCI) is crucial to facilitate early diagnosis and future clinical interventions. This study employed free‐water diffusion tensor imaging (FW‐DTI) to explore the differences in white matter alterations between EMCI and LMCI. Additionally, voxel‐based correlations between FW‐DTI metrics and Mini‐Mental State Examination (MMSE)/Geriatric Depression Scale (GDS) were studied.

**Methods:**

In this study, a total of 56 healthy controls (HCs) with a mean age <age> of 74.2 (±6.5) years, 43 individuals with EMCI with <age> of 72.1 (±8.1) years, and 28 subjects with LMCI with <age> of 73.8 (±6.0) years were included. Magnetic resonance imaging (MRI) data, acquired at 3T, were downloaded from the ADNI database. Pre‐processing steps included denoising, brain extraction, correction for eddy current, and alignment of diffusion data. FW‐DTI metrics were computed using a custom Matlab script. Statistical analysis was conducted using ANCOVA, employing a linear model with gender and age as covariates (significance for FDR<0.05). Linear models were also used for correlations.

**Results:**

Statistical differences between EMCI and LCMI were found for MMSE (Z=4.275;p<0.001). No differences were found for GD (Table 1). Figure 1 depicts group‐wise differences in the free‐water fraction (f) and FW‐Fractional Anisotropy (FW‐FA) across all study cohorts. Differences in the f index (f‐EMCI<f‐LMCI) were found in the forceps major, corpus callosum, and right fornix between EMCI and LMCI. Differences in FW‐FA metric (FW‐FA‐EMCI>FW‐FA‐LMCI) were observed in the forceps major, corpus callosum, right sagittal stratum, and right fornix, shedding light on specific white matter tracts implicated in the progression from early to late MCI stages. Voxel‐based correlations between FW‐DTI metrics and MMSE/GD are illustrated in Figure 2.

**Conclusions:**

In conclusion, our study reveals critical insights into the white matter changes associated with EMCI and LMCI, with significant implications for future research and clinical practice. The observed correlations between diffusion metrics and cognitive scores, mainly in LMCI group, underscore the potential of the FW‐DTI in enhancing our understanding of MCI progression and informing early diagnostic strategies.